# Comparative Determination of Mitochondrial Biomarkers and Their Relationship With Insulin Resistance in Type 2 Diabetic Patients: An Observational Cross‐Sectional Study

**DOI:** 10.1002/edm2.507

**Published:** 2024-06-28

**Authors:** Emmanuel K. Ofori, Wormenor Dziedzorm, Alfred Buabeng, Francis K. Dogodzi, Laurinda X. Adusu‐Donkor, Segla K. Bernard, Seth K. Amponsah, Henry Asare‐Anane

**Affiliations:** ^1^ Department of Chemical Pathology, UGMS University of Ghana Accra Ghana; ^2^ Paradise Diagnostic Centre Abeka‐Lapaz, Accra Ghana; ^3^ St. Gregory Hospital Buduburam Central Region Ghana; ^4^ School of Veterinary Medicine, College of Basic and Applied Sciences University of Ghana Accra Ghana; ^5^ 37 Military Hospital Accra Ghana; ^6^ West African Centre for Cell Biology of Infectious Pathogens Accra Ghana; ^7^ Department of Medical Pharmacology University of Ghana Medical School Accra Ghana

**Keywords:** acylcarnitine, citrate synthase, cytochrome c, mitochondria, type 2 diabetes

## Abstract

**Introduction:**

Data suggest malfunctioning mitochondria reduce oxidation and adenosine triphosphate (ATP) production, disrupting insulin signalling. Cytochrome c (CC), acylcarnitine (AC) and citrate synthase (CS) are essential components of the mitochondria machinery and can be used as reliable biomarkers of mitochondrial dysfunction. This study aimed to determine whether mitochondrial biomarkers (AC, CS and CC) are altered in individuals with type 2 diabetes mellitus (T2DM) and to examine the association between these biomarkers and insulin resistance.

**Methodology:**

A cross‐sectional observational study that recruited 170 participants (88 with T2DM and 82 without DM) was conducted. Blood samples were collected from the recruits and analysed for levels of fasting glucose (FBG), AC, CS, CC, insulin, total cholesterol, triglycerides (TG), glycated haemoglobin (HbA1c) and magnesium. Blood pressure (BP) and anthropometric characteristics of participants were also taken. Appropriate formulas were used to determine %body fat, body mass index (BMI), waist‐to‐hip ratio (WHR), the homeostatic model assessment for insulin resistance (HOMA‐IR) and insulin sensitivity (HOMA‐β).

**Results:**

Patients with T2DM had higher levels of CC, %body fat, FBG, TG, HbA1c, BMI and HOMA‐IR than controls (*p* < 0.05, respectively). Results showed a significant relationship between circulating CC levels versus HOMA‐β (*r* = −0.40, *p* = 0.001), CS (*r* = −0.70, *p* = 0.001) and AC (*r* = −0.72, *p* = 0.001) levels in patients with T2DM. The adjusted odds increased in the T2DM patients for VLDL (OR = 6.66, *p* = 0.002), HbA1c (OR = 6.50, *p* = 0.001), FPG (OR = 3.17, *p* = 0.001), TG (OR = 2.36, *p* = 0.010), being female (OR = 2.09, *p* = 0.020) and CC (OR = 1.14, *p* = 0.016).

**Conclusion:**

Overall, alterations in mitochondrial biomarkers, measured by AC, CC and CS, were observed in people with T2DM and showed a direct relationship with insulin resistance. These findings are potentially significant in Africa, although additional confirmation from a larger cohort is necessary.

## Graphical abstract

The standard multiple regression model, which incorporated mitochondrial biomarkers such as cytochrome c, acylcarnitine and citrate synthase, accounted for 72.2% of the variability in insulin resistance (HOMA‐IR). This finding emphasises the significant impact of mitochondrial dysfunction on insulin resistance and diabetes.

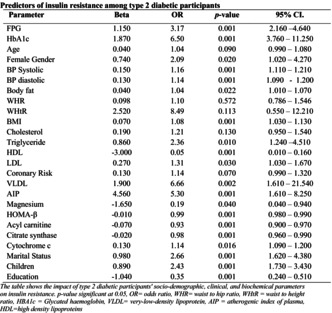

## 1 Introduction

Diabetes mellitus (DM) affects more than 400 million people worldwide, out of which approximately 1.6 million deaths occur annually [1]. Type 2 diabetes mellitus (T2DM) is anticipated to become widespread in Africa due to urbanisation, sedentary lifestyles and lack of physical activity [2, 3]. DM affects the socio‐economic status of patients, especially those from the sub‐Saharan region [4]. Thus, preventive measures to reduce incidence are paramount.

Mitochondria are the principal energy sources for cellular function and survival via oxidative phosphorylation. The mitochondrial matrix supports a variety of metabolic pathways, including fatty acid oxidation, the Krebs cycle, and lipid and cholesterol production. The literature shows that diminished mitochondrial function can decrease beta‐oxidation and adenosine triphosphate (ATP) synthesis. When it comes to glucose metabolism in adults, mitochondrial activity is an essential factor, with alterations in mitochondrial function being linked to insulin resistance and diabetes in both animals and humans [5, 6]. Nevertheless, it remains uncertain whether mitochondrial dysfunction is a necessary condition for the occurrence of these clinical conditions. Under resource‐constrained conditions, such as in sub‐Saharan Africa, the recommended use of a biopsy of the tissue of interest to evaluate mitochondrial DNA (mtDNA), its variations and the mtDNA genome might not always be possible. Therefore, alternative methods are necessary to ascertain the impact of mitochondrial dysfunction on different disease states. Using blood markers as a surrogate for this specific purpose is a top priority because of its convenience and easy accessibility. These biomarkers or enzymes used to evaluate mitochondrial dysfunction include acylcarnitine (AC), cytochrome c (CC) and citrate synthase (CS).

AC facilitates the transportation of long‐chain fatty acids across the inner mitochondrial membrane, aiding oxidation and subsequent production of ATP. Carnitine eliminates acyl residues and regulates coenzyme A, which preserves the integrity of the mitochondrial membrane [7, 8]. CS is an essential enzyme, which converts acetyl‐CoA and oxaloacetate into citrate and CoA. This reaction is irreversible. CS is thus identified as the rate‐limiting step in the tricarboxylic acid (TCA) cycle.

Consequently, this enzyme has been utilised in research to evaluate mitochondrial activity, content and function [9, 10]. CC is located in the inner membrane of the mitochondria and plays a crucial role in transferring electrons from complex III to complex IV. This electron transfer is essential for the mitochondrial energy metabolism pathway. CC is liberated in the event of mitochondrial malfunction [11]. These biomarkers can be easily measured using urine or serum samples instead of invasive biopsy and the more sophisticated mitochondrial DNA (mtDNA) sequencing assay. Despite the aforementioned, there have been conflicting results on the use of biomarkers of mitochondrial dysfunction in the diagnosis of metabolic disorders [12, 13]. This study aimed to assess AC, CS and CC levels as biomarkers for mitochondrial dysfunction and evaluate their relationship with IR in individuals with T2DM in Ghana.

## 2 Materials and Methods

### 2.1 Study Design, Site and Participants

This observational cross‐sectional study was conducted from March to July 2021 at the Lapaz Community Hospital, Accra, Ghana. The Lapaz Community Hospital is an accredited medical facility with two branches in Accra. It provides general and specialised services for individuals, particularly within the greater Accra region of Ghana. One hundred and seventy (170) participants were enrolled: 88 had T2DM and 82 were controls (without diabetes mellitus). T2DM individuals are those who have been diagnosed with diabetes between the ages of 20 and 70 and are using oral drugs to lower their blood sugar levels. Nondiabetic volunteers undergoing medical examination and staff with no history of type 2 diabetes had a fasting plasma glucose level below 6.1 mmol/L, and glycated haemoglobin levels below 5.7% [14] were recruited as controls. Before being recruited, participants were obligated to complete an informed consent form indicating their voluntary agreement to participate in the study. Before signing their consent forms, participants were provided with information regarding the purpose and duration of the study, the advantages of participating, the materials utilised for sample collection and the potential hazards associated with the sampling approach. Those who had misgivings or declined to participate in the study could get the usual treatment at the hospital. The study excluded individuals with tuberculosis, immune‐mediated disorders, immunocompromised people, those with degenerative diseases, pregnant and nursing mothers, patients having COVID‐19 therapy and people receiving insulin for diabetes management. Patients who had a severe condition that required immediate medical attention, such as severe chest pain, altered mental status, breathing difficulties and persistent bleeding, were also excluded from the study.

For minimum sample size determination, we established that 64 participants for each study group were adequate for this investigation. We used a 6.46% prevalence rate for T2DM in Ghana [15] at a 95% confidence interval and assumed a marginal error of 5%. A random sampling method was used, where an initial random volunteer was chosen from each stratum. Subsequently, every alternate volunteer was incorporated until the target sample size for each stratum was attained. The Ethical and Protocol Review Committee (EPRC) of the University of Ghana's College of Health Sciences granted approval for this study (ID: CHS‐Et/M.3‐5/2020‐2021). Further approval was sought from the management of the Lapas Community Hospital.

### 2.2 Clinical Assessment and Laboratory Procedures

A standardised questionnaire was employed to collect demographic and clinical information of study participants, encompassing their age, weight, height, educational background, diagnosis, duration, family history of DM and history of drug use. Waist circumference (WC) and hip circumference (HC) were measured using a body tape measuring tool. The body mass index (BMI) was calculated by dividing weight in kilograms (kg) by the height in square meters (kg/m^2^) of participants. The waist‐to‐height ratio was calculated by dividing the waist circumference by the height, measured in centimetres. The waist‐to‐hip ratio was calculated by dividing the measurement of the WC by the measurement of the HC. The body fat percentage was calculated using the subsequent formula. The formula applied to men was 0.567 × WC + 0.101 × age (years) – 31.8, while for females, the formula was 0.438 × WC + 0.221 × age (years) – 9.4 [16, 17]. After 20 min of rest, the participant's blood pressure (BP) was measured in the left arm using an automated BP monitor (OMRON Healthcare Company Limited, Kyoto, Japan) while maintaining a seated position. An average of two measurements was documented.

In the morning, between 7:00 and 9:00 a.m., each patient underwent venepuncture to obtain six millilitres (6 mL) of blood after abstaining from food for 10–14 h. A volume of 1 mL of whole blood was aliquoted into tubes containing sodium fluoride for glucose measurement. An additional volume of 2 mL was aliquoted into tubes containing EDTA for glycated haemoglobin (HbA1c) determination. The remaining 3 mL of blood was put into gel separator tubes and allowed to clot for 10–20 min. Subsequently, these clotted samples were centrifuged at 2000 rpm for 10 min. The resulting sera were stored at −20°C for roughly a month until analysed. CC, CS, AC and insulin levels were evaluated in sera using the enzyme‐linked immunosorbent assay according to the manufacturer's protocol (Sunlong Biotech Co., Ltd, China). The test utilises a sandwich‐type enzyme immunoassay that requires the binding of a double‐specific monoclonal antibody. The absorbances (OD) at 450 nm were measured using a microtitre plate reader (Varioskan lux, Thermo Fisher Scientific, USA), with the blank control's OD value and the reference point set to zero. The software Adamsel (RemaqueR) was utilised to transform the optical density measurements into concentrations. The levels of HbA1c in samples were assayed using fluorescence immunoassay (ichorma) according to the manufacturer's protocol (Boditech Med Inc., Republic of Korea). An automated chemistry analyser (Humastar 200, Human Diagnostics, Germany) was used to measure fasting plasma glucose (FPG), lipids profile and magnesium concentrations in blood. Alongside patient samples, quality control materials are assessed to confirm the precision and dependability of the employed analysers. The homeostatic assessment for insulin resistance (HOMA‐IR) and beta cell function (HOMA‐β) were calculated using previously reported formulas [18, 19]. Low‐density lipoprotein (LDL) cholesterol was derived using Frieldewald's equation [20]. The coronary risk was calculated by dividing the total cholesterol by the HDL cholesterol.

### 2.3 Statistical Analysis

Data were initially summarised using Microsoft Excel (2010). The Statistical Package for the Social Sciences (SPSS) version 21.0 was employed for subsequent statistical analysis. Data were presented as descriptive statistics (mainly tables), and the data's normality was assessed using the Shapiro–Wilk test. The clinical and biochemical parameters of the participants with T2DM were compared to those without DM using an independent‐sample *t*‐test. Pearson's product–moment correlation coefficient (*r*) and the binary logistic regression model were used to assess the influence of sociodemographic, clinical and biochemical variables on HOMA‐IR. A *p* value below 0.05 was deemed statistically significant.

## 3 Results

The study included 88 individuals with confirmed T2DM as the case group and 82 healthy individuals without DM as the control group. The majority of participants in both groups were female (81.8% in cases and 68.3% in control), married (62.5% in cases and 68.3% in control), had more than one child (81.8% in cases and 56.2% in control), identified as Christians (85.2% in cases and 93.9% in control) and reported not drinking alcohol (78.4% in cases and 78.0% in control). Most persons in the case group (47.8%) spoke the local dialect Twi, while in the control group, individuals who spoke English constituted the dominant group (50.0%). In the case group, 56.8% of the participants stated they were self‐employed, compared to 41.4% in the control group. Table 1 summarises the sociodemographic characteristics of the participants.

**TABLE 1:** Sociodemographic and clinical characteristics of the study participants.

Variables	Cases *N* = 88 (%)	Control *N* = 82 (%)	Chi‐square (*χ* ^2^)	*p*
*Gender*				
Male	16 (18.2)	26 (31.7)	4.174	0.040
Female	72 (81.8)	56 (68.3)		
*Marital status*				
Single	4 (4.5)	17 (20.7)	31.788	0.001
Married	55 (62.5)	56 (68.3)		
Divorced	16 (18.2)	6 (7.3)		
Separated	2 (2.3)	2 (2.4)		
Widowed	11 (12.5)	1 (1.2)		
*Educational status*				
Primary	19 (21.6)	2 (2.4)	20.138	0.001
JHS/middle school	38 (43.2)	18 (22.0)		
SHS/‘O’ level	16 (18.2)	26 (31.7)		
Tertiary	15 (17.0)	36 (43.9)		
*Employment status*				
Self‐employed	50 (56.8)	34 (41.4)	43.681	0.001
Employed	22 (25.0)	47 (57.3)		
Retired	16 (18.2)	1 (1.2)		
*Number of children*				
None	5 (5.7)	4 (4.9)	26.597	0.001
One	4 (4.5)	32 (39.0)		
Two	26 (29.5)	30 (36.6)		
Three	30 (34.1)	13 (15.9)		
Four	16 (18.2)	3 (3.7)		
*Languages spoken*				
English	35 (39.8)	41 (50.0)	1.944	0.579
Twi	42 (47.7)	33 (40.2)		
Ewe	9 (10.2)	6 (7.3)		
Ga	2 (2.3)	2 (2.4)		
*Religion*				
Christian	75 (85.2)	77 (93.9)	3.374	0.069
Muslim	13 (14.8)	5 (6.1)		
*Alcohol intake*				
Yes	19 (21.6)	18 (22.0)	0.011	0.94
No	69 (78.4)	64 (78.0)		
*Family history DM*				
Yes	57 (64.8)	45 (54.9)	1.732	0.188
No	31 (35.2)	37 (45.1)		

*Note:* The sociodemographic characteristics of the study participants.

Abbreviations: DM, diabetes mellitus; JHS, junior high school; *N*, frequency; SHS, senior high school; %, percentages.

While there was no significant difference in age and WC between the case group and the controls (*p* > 0.05), the case group exhibited higher BMI, %body fat and blood pressure measures than the control group (*p* < 0.05, respectively). Furthermore, the case group demonstrated higher FPG, HbA1c, HOMA‐IR, TG, LDL, VLDL and CC levels than the controls (*p* < 0.05 for all). In contrast, the control group exhibited significantly higher levels of insulin, HOMA‐β, magnesium, AC and CS levels than the case group (*p* < 0.05 for each) (Table 2).

**TABLE 2:** Clinical (continuous) and biochemical parameters of the study participants.

Features	Cases	Control	95% CI	*p*
	Mean ± SD	Mean ± SD		
*Clinical parameters*				
Age (years)	52.03 ± 7.88	51.30 ± 7.80	48.52–51.00	0.090
Weight (kg)	79.40 ± 20.36	71.98 ± 13.55	73.07–78.56	0.013
Height (m^2^)	1.60 ± 0.09	1.64 ± 0.08	1.60–1.63	0.019
BMI (kg/m^2^)	31.26 ± 8.92	27.18 ± 6.49	28.07–30.52	0.001
Systolic blood pressure (mmHg)	133.28 ± 16.0	117.34 ± 6.60	123.37–127.82	0.001
Diastolic blood pressure (mmHg)	88.58 ± 11.02	78.72 ± 6.78	82.24–85.40	0.001
% Body fat	43.33 ± 9.7	39.64 ± 9.99	40.04–43.06	0.020
Waist circumference (cm)	100.59 ± 14.96	98.98 ± 13.76	97.64–101.99	0.471
Hip circumference (cm)	112.57 ± 16.36	113.01 ± 17.58	110.22–115.34	0.869
Waist‐to‐hip ratio	0.90 ± 0.06	0.88 ± 0.07	0.88–0.90	0.157
*Metabolic parameters*				
FPG (mmol/L)	10.75 ± 4.42	5.32 ± 0.87	7.49–8.77	0.001
HbA1c (%)	7.57 ± 2.07	4.85 ± 0.74	5.94–6.7	0.001
Insulin	9.01 ± 5.76	12.58 ± 5.73	9.82–11.64	0.008
HOMA‐IR	3.89 ± 2.31	2.99 ± 1.56	3.14–3.76	0.009
HOMA‐β	58.43 ± 104.58	182.70 ± 151.43	96.69–140.05	0.001
Magnesium (mmol/L)	2.18 ± 0.18	2.24 ± 0.21	2.18–2.24	0.042
*Lipid parameters*				
Total cholesterol (mmol/L)	5.62 ± 1.25	5.31 ± 1.29	5.28–5.66	0.124
Triglycerides (mmol/L)	1.40 ± 0.80	1.13 ± 0.50	1.17–1.38	0.012
HDL (mmol/L)	1.03 ± 0.27	1.36 ± 0.32	1.11–1.20	0.007
LDL (mmol/L)	3.94 ± 1.14	3.49 ± 1.22	3.53–3.89	0.026
VLDL (mmol/L)	0.64 ± 0.36	0.51 ± 0.23	0.53–0.63	0.013
Coronary risk (ratio)	5.01 ± 1.04	4.39 ± 1.69	4.70–5.12	0.062
*Mitochondrial markers*				
Acyl carnitine (μmol/L)	30.29 ± 8.86	37.46 ± 11.98	32.07–35.42	0.001
Citrate synthase (mg/mL)	40.01 ± 20.25	50.65 ± 22.79	41.80–48.50	0.001
Cytochrome c (ng/mL)	24.46 ± 11.86	13.16 ± 6.74	17.31–20.71	0.001

*Note:* A comparison of levels of biochemical and clinical (continuous) parameters of the study participants. Data presented as mean. A *p* value < 0.05 was considered significant.

Abbreviations: BMI, body mass index; FPG, fasting plasma glucose; HbA1c, glycated haemoglobin; HDL, high‐density lipoprotein cholesterol; HOMA‐IR, insulin resistance; HOMA‐β, Insulin sensitivity; LDL, low‐density lipoprotein cholesterol; SD, standard deviation; VLDL, very‐low‐density lipoprotein.

Pearson's product–moment correlation analysis was performed to establish relationships between the mitochondrial biomarkers and other clinical and biochemical indicators among participants in both groups. The clinical parameters, namely BMI, WC and %body fat, showed a negative correlation with AC (*r* = −0.82; −0.63; −0.42; *p* < 0.05 for all) and CS (*r* = −0.79; −0.64; −0.34; *p* < 0.05 for all), but a positive correlation with CC (*r* = 0.73; 0.57; 0.46; *p* < 0.05 for all) in the case group (Table 3). While HOMA‐β, AC and CS levels were associated negatively with CC (*r* = −0.40; −0.72; −0.70; *p* < 0.05 for all), positive associations were observed between CC versus total cholesterol, TG, LDL, coronary risk, VLDL and AIP (*r* = 0.21; 0.24; 0.22; 0.27; 0.35; 0.41; *p* < 0.05 for all) (Table 3). The standard multiple regression model, including mitochondrial biomarkers, explained 72.2% (*R* ^2^ = 0.783; Adj. *R* ^2^ = 0.722; *p* < 0.001) of the variance in HOMA‐IR levels among the case group. Significant contributions were made by VLDL (OR = 6.66, *p* = 0.002), HbA1c (OR = 6.50, *p* = 0.001), FPG (OR = 3.17, *p* = 0.001), being married (OR = 2.66, *p* = 0.001), TG (OR = 2.36, *p* = 0.010), female gender (OR = 2.09, *p* = 0.020) and CC (OR = 1.14, *p* = 0.016) (Table 4) in the case group.

**TABLE 3:** Relationship between several correlates with insulin resistance, β‐cell function and mitochondria biomarkers among the case group.

Parameter	HOMA‐IR		HOMA‐B		AC		CS		CC	
	*r*	*p*	*r*	*p*	*r*	*p*	*r*	*p*	*r*	*p*
BMI	0.11	0.173	−0.11	0.243	−0.82	0.001	−0.79	0.001	0.73	0.001
WC	−0.05	0.538	−0.14	0.361	−0.63	0.001	−0.64	0.001	0.57	0.001
HC	−0.05	0.559	−0.04	0.648	−0.53	0.001	−0.61	0.001	0.46	0.001
Body fat	0.02	0.886	−0.14	0.076	−0.42	0.001	−0.34	0.005	0.30	0.007
WHR	−0.02	0.787	−0.10	0.062	0.10	0.314	−0.13	0.188	−0.06	0.647
WHtR	−0.07	0.396	−0.21	0.040	−0.67	0.001	−0.58	0.001	0.60	0.001
Glucose	0.29	0.007	−0.53	0.002	−0.11	0.649	0.08	0.689	0.09	0.417
HBA1c	0.23	0.016	−0.61	0.001	−0.24	0.013	−0.15	0.062	0.14	0.061
Total cholesterol	0.26	0.020	−0.20	0.041	−0.11	0.178	−0.11	0.071	0.21	0.042
Triglyceride	0.10	0.351	0.28	0.040	0.28	0.003	0.23	0.014	0.24	0.013
HDL	0.16	0.148	−0.13	0.071	0.11	0.287	0.06	0.747	0.09	0.652
LDL	0.04	0.716	−0.21	0.042	0.20	0.039	−0.12	0.053	0.22	0.037
Coronary risk	0.20	0.011	−0.20	0.042	−0.11	0.166	−0.19	0.034	0.27	0.008
VLDL	0.21	0.043	−0.32	0.013	−0.12	0.061	0.24	0.018	0.35	0.006
AIP	0.30	0.004	−0.79	0.001	−0.16	0.058	−0.22	0.033	0.41	0.002
Magnesium	−0.15	0.187	0.08	0.77	−0.11	0.328	−0.06	0.538	0.13	0.197
Insulin	0.73	0.001	0.66	0.001	0.07	0.689	0.09	0.324	−0.18	0.032
HOMA‐IR	1.00		0.10	0.203	−0.13	0.061	−0.09	0.286	0.11	0.094
HOMA‐β	0.10	0.706	1.00		0.19	0.040	0.13	0.062	−0.40	0.001
Acyl carnitine	−0.18	0.413	0.11	0.140	1.00		0.74	0.001	−0.72	0.001
Citrate synthase	−0.19	0.278	0.16	0.041	0.74	0.001	1.00		−0.70	0.001
Cytochrome c	0.13	0.481	−0.40	0.002	−0.72	0.001	−0.70	0.001	1.00	

*Note: p* value significant at 0.05.

Abbreviations: AIP, atherogenic index of plasma; BMI, body mass index; DL, high‐density lipoprotein; HbA1c, glycated haemoglobin; HOMA‐IR, insulin resistance; HOMA‐β, insulin sensitivity; LDL, low‐density lipoprotein; *r*, Pearson's correlation coefficient; VLDL, very‐low‐density lipoprotein; WC, waist circumference; WHR, waist‐to‐hip ratio; WHtR, waist‐to‐height ratio.

**TABLE 4:** Predictors of insulin resistance among the case participants.

Parameter	*β*	OR	*p*	95% CI
FPG	1.150	3.17	0.001	2.160–4.640
HbA1c	1.870	6.50	0.001	3.760–11.250
Age	0.040	1.04	0.090	0.990–1.080
Female gender	0.740	2.09	0.020	1.020–4.270
BP systolic	0.150	1.16	0.001	1.110–1.210
BP diastolic	0.130	1.14	0.001	1.090–1.200
Body fat	0.040	1.04	0.022	1.010–1.070
WHR	0.098	1.10	0.572	0.786–1.546
WHtR	2.520	8.49	0.113	0.550–12.210
BMI	0.070	1.08	0.001	1.030–1.130
Cholesterol	0.190	1.21	0.130	0.950–1.540
Triglyceride	0.860	2.36	0.010	1.240–4.510
HDL	−3.000	0.05	0.001	0.010–0.160
LDL	0.270	1.31	0.030	1.030–1.670
Coronary risk	0.130	1.14	0.070	0.990–1.320
VLDL	1.900	6.66	0.002	1.610–21.540
AIP	4.560	5.30	0.001	1.610–8.250
Magnesium	−1.650	0.19	0.040	0.040–0.940
HOMA‐β	−0.010	0.99	0.001	0.980–0.990
Acyl carnitine	−0.070	0.93	0.001	0.900–0.970
Citrate synthase	−0.020	0.98	0.001	0.960–0.990
Cytochrome c	0.130	1.14	0.016	1.090–1.200
Marital status	0.980	2.66	0.001	1.620–4.380
Children	0.890	2.43	0.001	1.730–3.430
Education	−1.040	0.35	0.001	0.240–0.510

*Note:* The impact of the case participants' sociodemographic, clinical and biochemical parameters on their HOMA‐IR levels. *p* value significant at 0.05.

Abbreviations: AIP, atherogenic index of plasma; HBA1c, glycated haemoglobin; HDL, high‐density lipoproteins; OR, odds ratio; VLDL, very‐low‐density lipoprotein; WHR, waist‐to‐hip ratio; WHtR, waist‐to‐height ratio.

## 4 Discussion

This study was conducted to determine whether or not persons in Ghana diagnosed with T2DM had altered levels of mitochondrial biomarkers (AC, CS and CC) and evaluate the link between these biomarkers and insulin resistance. When studying the association between ageing and indicators of mitochondrial dysfunction, we found that age correlated negatively with AC and CS levels. In contrast, we found a positive correlation between age and CC levels. Ageing is characterised by a steady decline in metabolic and physiological processes, which eventually can lead to morbidity and mortality. In ageing, there is a decrease in energy expenditure, resulting in a reduction in ATP requirements because of the regulation of energy production. As a result, animals and humans experience reduced oxidative capacity in their skeletal muscles and heart. There seem to be many apoptotic signals that can cause mitochondria to release biomolecules that induce cell death, such as CC. CC, a mitochondrial proapoptotic agent, is essential for transferring electrons from Complex III to Complex IV in the electron transport chain, plays a role in initiating apoptosis, a mechanism of controlled cell death [21] and has been linked to the progression of mitochondrial dysfunction and IR [22]. One important step in triggering cell death could be the release of these CCs into the bloodstream. As one age, there is also an increase in free radical and reactive oxygen species formation, causing oxidative stress, systemic inflammation and mitochondrial failure, eventually resulting in IR [5, 22].

Previous studies on the influence of AC and CS on mitochondrial function have produced conflicting findings. Some have found reduced CS levels in diabetic human myocytes but no change in carnitine levels between type 2 diabetics and nondiabetics [23, 24, 25]. One study found reduced levels of carnitine in individuals with complicated diabetes compared to those with uncomplicated diabetes [26]. Reduced levels of carnitine have been observed in T2DM patients with cardiovascular disease [27]. In addition, a 6‐month oral supplementation of AC did not impact glycaemia, insulin sensitivity and kidney function in T2DM patients with hypertension and receiving statin medication [28]. Possible causes of lower AC and CS levels seen in this study could be because of ageing, diminished mitochondrial function, dietary restrictions, decreased kidney reabsorption, a malfunction in the enzyme itself and diabetic complications. Diabetic individuals commonly have reduced levels of AC and CS as a result of dietary limitations intended to lessen the impact of diabetes‐related issues. The restrictions have a detrimental effect on the consumption of essential nutrients, especially carnitine. Indeed, the positive impact of calorie restriction, achieved through the expansion of mitochondrial biogenesis and function, can improve glucose and lipid metabolism, and insulin resistance [29]. Therefore, therapeutic strategies to increase mitochondrial function or biogenesis may decrease insulin resistance and other aspects of the metabolic syndrome. A malfunction of the AC and CS genes might hinder the complete oxidation of fatty acids in muscle, leading to insulin resistance [30, 31].

Carnitine safeguards cell membrane integrity and maintains the balance between coenzyme A and acetyl‐CoA in mitochondria. Several studies have proposed l‐carnitine supplementation in diabetes mellitus management because of its assistance in transporting long‐chain fatty acids across the mitochondrial membrane [32, 33]. The discovery that supplementing with carnitine increases glucose tolerance while increasing the presence of acylcarnitines in the bloodstream supports the idea that the production and release of these proteins are advantageous rather than harmful. Therefore, we consider these metabolites as indicators rather than agents that cause metabolic disorders.

This study also examined the association between body composition, metabolic parameters and biomarkers related to mitochondrial dysfunction. We observed a negative association between AC and CS levels versus BMI, %body fat, WHtR and WHR (Table 3). We further observed lower levels of AC and CS versus higher levels of total cholesterol and triglycerides in individuals with diabetes. In contrast, positive correlations were found for body composition characteristics, total cholesterol, triglycerides and coronary risk versus CC levels (Table 3). Obesity has an important role in triggering the development of T2DM. Although not fully understood, CS activity shortage may have a role in the onset of T2DM, leading to higher body fat levels [24]. In diabetes, there is an elevation in reactive oxygen species generation and a decrease in the efficiency of the mitochondrial respiratory chain [34, 35]. This increase in CC levels may be because of increased mitochondrial damage caused by diabetes. Elevated blood lipid levels with CC may suggest mitochondrial malfunction and increased lipidemia [36]. Research conducted on lymphocytes from obese individuals found elevated CC levels, which were attributed to ageing and obesity [37, 38].

In the current study, the standard multiple regression model, including mitochondrial biomarkers, explained 72.2% of the variation in IR (HOMA‐IR) (Table 4), highlighting the important role of mitochondrial dysfunction in causing IR and diabetes. Identifying CC as a key factor in IR underscores the need to focus on mitochondrial function in managing diabetes. By clarifying these associations, our study establishes a basis for creating new therapeutic methods to maintain mitochondrial health and enhance insulin sensitivity in diabetes individuals. Evaluating CC levels may help to identify high‐risk people who are not identifiable using conventional clinical tests. There are notable disparities between men and women regarding insulin resistance, body composition and energy balance. The quantity, composition and distribution of particular adipose tissue have been found to have a detrimental impact on insulin sensitivity. This could perhaps account for the higher level of IR observed in females as opposed to males. Furthermore, since most individuals in this cohort were female and married, we can attribute these substantial findings to our study participant characteristics or random chance.

This study had limitations; the cross‐sectional approach limits the determination of causal links between the variables studied, highlighting the necessity for longitudinal studies. Additional factors, such as dietary habits and levels of physical activity, were not taken into consideration. There is a possibility that other lipid components not measured in this study, such as ceramide and diacylglycerol, may have a stronger relationship with these indicators of mitochondrial metabolism. The generalisability of our findings may be limited by the unique characteristics of our study sample, requiring validation in larger cohorts. A hyperinsulinemic–euglycemic clamp approach to measuring glucose disposal rates would have been optimal for this investigation, as higher basal insulin levels may not always indicate an issue.

## 5 Conclusion

In summary, individuals with type 2 diabetes showed changes in mitochondrial indicators as measured by AC, CC and CS. These biomarker changes further demonstrated a direct connection with insulin resistance. However, further validation from a larger sample group is necessary to ascertain the potential importance of these findings in Africa.

## Author Contributions

**Emmanuel K. Ofori** was involved in conceptualisation, supervision, writing–original draft and writing–review and editing. **Wormenor Dziedzorm** was involved in conceptualisation, methodology, investigation and writing–original draft. **Alfred Buabeng, Laurinda X. Adusu‐Donkor, Francis K. Dogodzi** and **Segla K. Bernard** were involved in methodology, investigation and resources. **Seth K. Amponsah** was involved in software and writing–review and editing. **Henry Asare‐Anane** was involved in supervision and writing–review and editing.

## Ethics Statement

The Ethical and Protocol Review Committee of the College of Health Sciences, University of Ghana, approved this study (ID: CHS‐Et/M.3‐5/2020‐2021). Before their enrolment in the study, all participants provided written informed consent. The research participants were thoroughly briefed on the potential hazards and benefits associated with their involvement in the study.

## Conflicts of Interest

The authors declare no conflicts of interest.

## Data Availability

The data that support the findings of this study are available from the corresponding author upon reasonable request.
